# Improved anti-glioblastoma efficacy by IL-13Rα2 mediated copolymer nanoparticles loaded with paclitaxel

**DOI:** 10.1038/srep16589

**Published:** 2015-11-16

**Authors:** Baoyan Wang, Lingyan Lv, Zhi Wang, Yan Jiang, Wei Lv, Xin Liu, Zhongyuan Wang, Yue Zhao, Hongliang Xin, Qunwei Xu

**Affiliations:** 1 Department of Pharmaceutics, School of Pharmacy, Nanjing Medical University, Nanjing 211166, China; 2 Nanjing Drum Tower Hospital. The Affiliated Hospital of Nanjing University Medical School. Nanjing 210008, China

## Abstract

Glioma presents one of the most malignant brain tumors, and the therapeutic effect is often limited due to the existence of brain tumor barrier. Based on interleukin-13 receptor α2 (IL-13Rα2) over-expression on glioma cell, it was demonstrated to be a potential receptor for glioma targeting. In this study, Pep-1-conjugated PEGylated nanoparticles loaded with paclitaxel (Pep-NP-PTX) were developed as a targeting drug delivery system for glioma treatment. The Pep-NP-PTX presented satisfactory size of 95.78 nm with narrow size distribution. Compared with NP-PTX, Pep-NP-PTX exhibited significantly enhanced cellular uptake in C6 cells (p < 0.001). The *in vitro* anti-proliferation evaluation showed that the IC_50_ were 146 ng/ml and 349 ng/ml of Pep-NP-PTX and NP-PTX, respectively. The *in vivo* fluorescent image results indicated that Pep-NP had higher specificity and efficiency in intracranial tumor accumulation. Following intravenous administration, Pep-NP-PTX could enhance the distribution of PTX *in vivo* glioma section, 1.98, 1.91 and 1.53-fold over that of NP-PTX group after 0.5, 1 and 4 h, respectively. Pep-NP-PTX could improve the anti-glioma efficacy with a median survival time of 32 days, which was significantly longer than that of PTX-NP (23 days) and Taxol^**®**^ (22 days). In conclusion, Pep-NP-PTX is a potential targeting drug delivery system for glioma treatment.

Glioblastoma multiforme (GBM) accounts for 12–15% of all brain tumors and 50–60% of astrocytomas. The incidence of GBM is less than 10 to 100,000, but the median survival of a little over 1 year from diagnosis makes it one of the most frequent primary malignant brain tumors^1^. The conventional clinical treatment for GBM involves surgical debulking of the accessible tumor from the patient’s brain, but its diffuse invasion of the surrounding normal tissue makes the complete removal of tumor by the conventional surgical method very difficult and tumor recurrence from residual tumors very possible^2^,^3^.

Consequently, chemotherapy is essential for the treatment of GBM. Paclitaxel (PTX) is a potent anticancer agent by promoting microtubule assembly, leading to inhibition of cell proliferation and induction of apoptosis^4^. However, the anti-glioma effect of PTX is very limited and often causes systemic side effects^5^, mainly due to its poor permeability across the blood tumor barrier (BTB)^6^.

Receptor-mediated endocytosis is one of the mechanisms through which carriers can overcome the obstacle of BTB and simultaneously target brain tumor. It is widely known that tumor cells express cell surface molecules such as specific antigens or cytokine receptors^7^,^8^. The interleukin-13 receptor α2 (IL-13Rα2), one of the subunits of the interleukin-13 receptor, is a tumor-specific receptor which is overexpressed in GBM. Because of its nonsignaling function, IL-13Rα2 has no signaling box motifs and behaves as a decoy receptor^9^. It has been reported that IL-13Rα2 can undergo internalization after binding to ligand without causing activation of its signaling pathways^10^. Based on the high affinity with IL-13Rα2, Interleukin-13 (IL-13) has been used as a ligand in glioma trials of immunotoxin therapy for GBM^11^. IL13-PE38QQR, a conjugation complex of human IL-13 with a mutated form of pseudomonas exotoxin, can lead to pronounced antitumor activity in GBM^12^,^13^,^14^,^15^. These observations make IL-13 Rα2 attractive as a targeting moiety.

Nanoparticulate drug delivery systems have been attracted increasing attentions in recent years. The combination of polymer nanoparticle and receptor-mediated endocytosis could provide a non-invasive alternative platform due to its active targeting property besides the passive targeting by the enhanced permeability and retention (EPR) effects of tumor^16^. It was reported that Pep-1 (CGEMGWVRC) peptide could facilitate polymer nanoparticles targeting to glioma via IL-13Rα2 endocytosis^17^. However, the value of Pep-1 modified copolymer nanoparticle in glioma targeting chemotherapy is yet unknown. Therefore, the objective of this study was to investigate the potential and safety of Pep-1 conjugated copolymer nanoparticles for delivering PTX as the chemotherapeutic drug to the treatment of glioma. PTX-loaded PEG-PLGA nanoparticles modified with Pep-1 (Pep-NP-PTX) were developed by the emulsion/solvent evaporation method. For *in vitro* study, both the uptake and anti-proliferation effect of nanoparticles in C6 glioma cells were evaluated. The *in vivo* penetration ability across blood tumor barrier of Pep-NP-PTX was studied qualitatively and quantitatively. The *in vivo* anti-glioma efficacy of Pep-NP-PTX was investigated by intracranial glioma-bearing mice model. The preliminary safety of Pep-NP following intravenous injection was evaluated using healthy mice.

## Results

### Characterization of the Pep-NP-PTX

**Figure 1:**
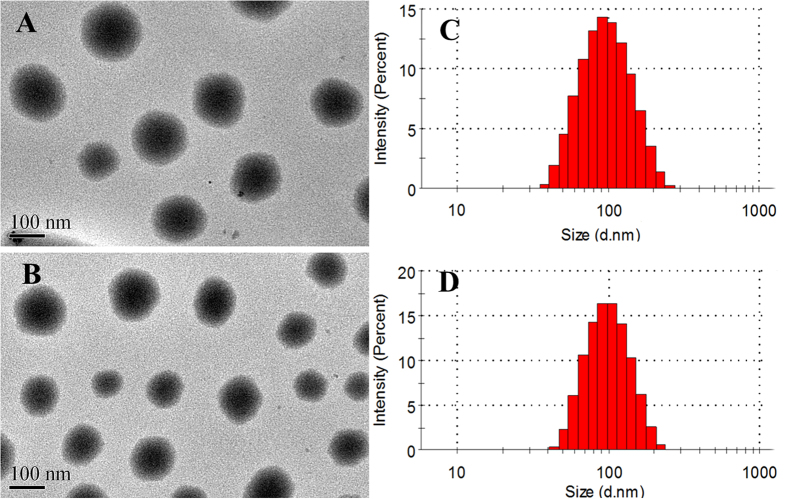
TEM image of NP-PTX (**A**) and Pep-NP-PTX (**B**); the size distribution of NP-PTX (**C**) and Pep-NP-PTX (**D**).

The nanoparticles were prepared via an emulsion/solvent evaporation method. The mean size of NP-PTX were 91.23 ± 1.56 nm (Fig. 1C), after modified with Pep-1 peptide, the size slightly increased to 95.78 ± 2.37 nm with the same narrow size distribution of Pep-NP-PTX (Fig. 1D). TEM photographs showed that NP-PTX and Pep-NP-PTX were generally spherical and of regular size (Fig. 1A,B), consistent with the size distribution. Due to the mean diameters less than 100 nm, such nanoparticles may accumulate more readily in tumor due to the EPR effect. Zeta potential of NP-PTX and Pep-NP-PTX were −35.2 ± 2.15 mV and −34.5 ± 1.74 mV, respectively, indicating that the surfaces of NP and Pep-NP were both strong negative charged (Table 1). The result suggested that the constructed nanoparticles had a good stability.

The encapsulation efficiency (EE) of NP-PTX and Pep-NP-PTX was 79.83 ± 1.51% and 77.27 ± 0.95%, respectively, with the loading capacity (LC) was 3.93 ± 0.096% and 3.77 ± 0.104%, respectively (Table 1).

### *In vitro* PTX release

**Figure 2:**
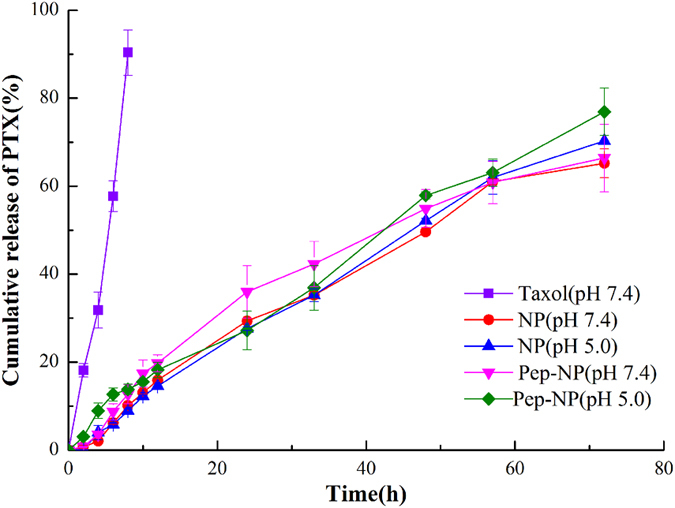
Paclitaxel release profiles from NP and Pep-NP in PBS (pH 5.0) and PBS (pH 7.4) containing 0.1% Tween-80.

The cumulative release curves of NP-PTX and Pep-NP-PTX under different pH conditions were presented in Fig. 2. Compared with Taxol^®^, NP-PTX and Pep-NP-PTX presented almost the same controlled release behavior. As showed in Fig. 2, 65.23% of PTX in NP and 66.43% of PTX in Pep-NP were released in PBS (pH 7.4), while 70.28% of PTX in NP and 76.94% of PTX in Pep-NP were released in PBS (pH 5.0) after 72 h. There was no significant difference about the release pattern between NP-PTX and Pep-NP-PTX in both pH 7.0 and pH 5.0. Therefore, the modification of Pep-1 peptide did not change the release pattern of PTX from the nanoparticles.

### Cellular uptake of PTX-loaded Pep-NP

**Figure 3:**
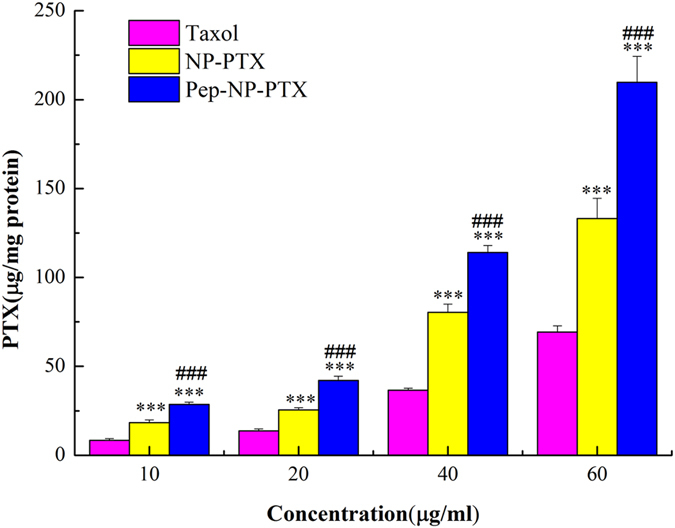
Cellular uptake of PTX-loaded NP and Pep-NP at 37 °C after incubation for 2 h at the PTX concentrations from 10 μg/mL to 60 μg/mL in C6 cells. Data represented mean ± SD (n = 3). ***p < 0.001 significantly higher than that of Taxol^®^, ###p < 0.001 significantly higher than that of NP-PTX.

The quantitative cellular uptake of PTX-loaded Pep-NP in C6 cells which was shown concentration-dependent model was presented in Fig. 3. From the results, it could be seen that the cellular uptake of Pep-NP group was 1.55, 1.65, 1.42 and 1.58 folds higher than that of NP group, and 3.39, 3.06, 3.11 and 3.03 folds higher than that of Taxol^®^ group at 37 °C at the PTX concentration of 10, 20, 40 and 60 μg/mL, respectively.

### Cell cytotoxicity

**Figure 4:**
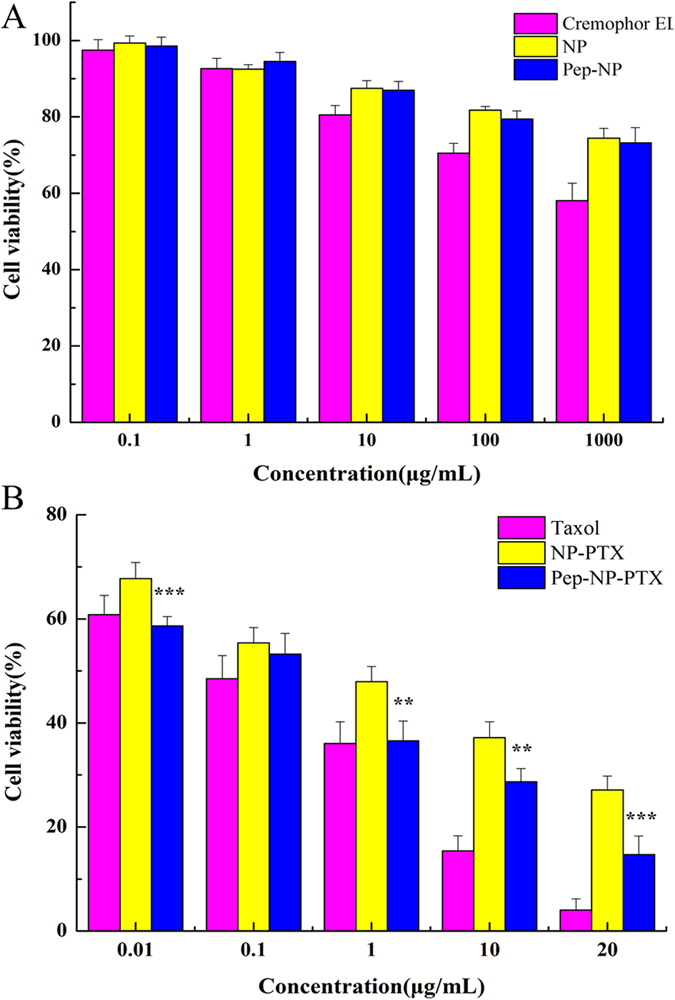
*In vitro* viability of C6 cells of Cremophor EL and blank nanoparticles at concentration ranging from 0.1 to 1000 μg/mL at 72 h (**A**); *In vitro* cytotoxicity of Taxol^®^ and PTX-loading nanoparticles at various concentrations at 72 h (**B**). Data represented mean ± SD (n = 3). **p < 0.01, ***p < 0.001 significantly lower than that of NP-PTX.

The *in vitro* cell cytotoxicity of different formulations was evaluated on C6 cells by using MTT method. As shown in Fig. 4A, the cytotoxicity of Cremophor EL was obviously higher than that of nanoparticles when the incubation concentration was more than 10 μg/mL. However, blank NP and Pep-NP did not show obvious cytotoxicity at concentrations ranged from 0.1 μg/mL to 1000 μg/mL. These results suggested that blank NP and Pep-NP were not toxic to C6 cells probably due to the biocompatibility of the block polymers.

The cell cytotoxicity of Taxol^®^, NP-PTX and Pep-NP-PTX in C6 cells showed a concentration-dependent behavior (Fig. 4B). Different degrees of cytotoxicity were found in all the PTX formulations. When compared with NP-PTX (IC_50_ values: 0.349 ± 0.012 μg/mL), Pep-NP-PTX exhibited much higher toxicity (IC_50_ values: 0.146 ± 0.002 μg/mL) at the concentrations ranged from 0.01 μg/mL to 20 μg/mL (Table 2).

### *In vivo* imaging

**Figure 5:**
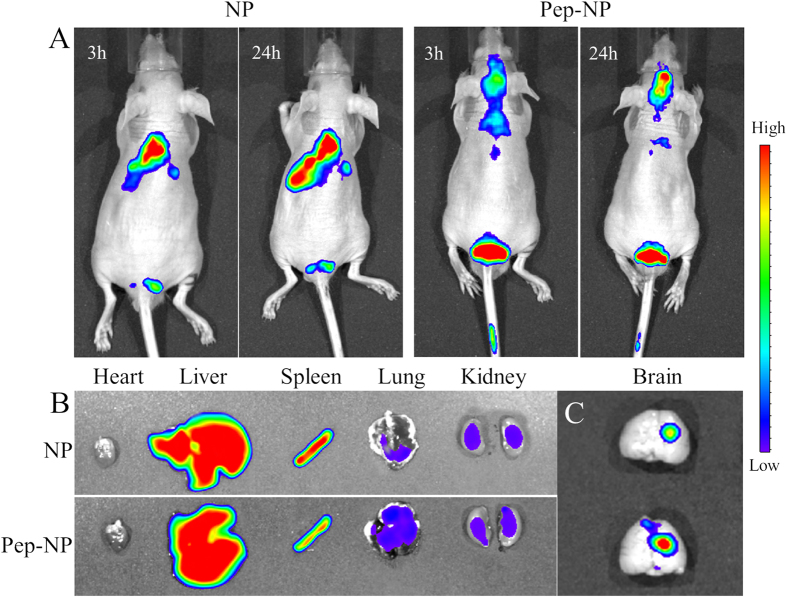
*In vivo* fluorescence imaging of intracranial C6 glioma-bearing nude mice after intravenous injection of Dir-labeled nanoparticles (**A**).Image of the tissues (**B**) and brains (**C**) sacrificed 24 h after intravenous injection of nanoparticles.

The *in vivo* glioma-targeting efficiency of NP and Pep-NP was also investigated in intracranial C6 glioma-bearing nude mice. Compared with NP group, the fluorescence intensity of Pep-NP group in the glioma site was much stronger than NP group at any time post-injection (Fig. 5A).

After 24 h post-injection, the fluorescence of tissues (heart, liver, spleen, lung, kidney and brain) was also visualized under the *in vitro* imaging system. As shown in Fig. 5B, intensive fluorescence signal was observed in liver and spleen, indicating that most of the nanoparticles were nonspecifically taken up and eliminated by mononuclear phagocyte system (MPS). However, *ex vivo* evaluation of brains showed an obvious glioma site accumulation of the nanoparticles (Fig. 5C). The fluorescence of Pep-NP group was much stronger than that of NP group in the glioma section, indicating that Pep-1 peptide could facilitate the enrichment of nanoparticles in the glioma via IL-13Rα2 mediated endocytosis.

### Brain biodistribution

Brain biodistribution of PTX following intravenous administration of Taxol^®^, NP-PTX and Pep-NP-PTX were assessed in intracranial C6 glioma-bearing mice. The amount of PTX in the normal brain and glioma section was determined with HPLC-mass spectrometry. There was no significant difference between the three formulations in the normal brain tissue at any time points post-injection.

**Figure 6:**
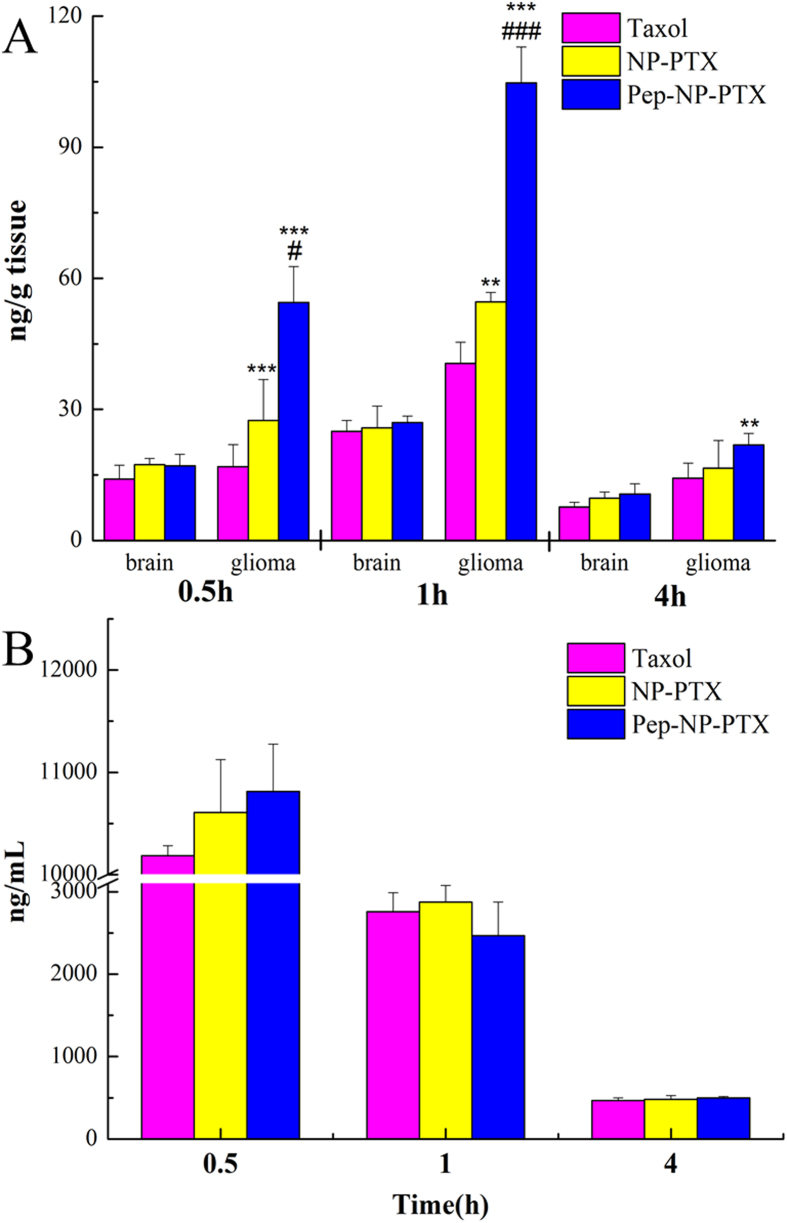
The PTX concentration of Taxol^®^, PTX-loaded NP and Pep-NP in brain and glioma (**A**), in plasma (**B**) at different times following i.v. administration to intracranial glioma-bearing ICR mice at a single 8 mg/kg dose of PTX (n = 5). **p < 0.01, ***p < 0.001 significantly higher than that of Taxol^®^, #p < 0.05, ###p < 0.001 significantly higher than that of NP-PTX.

At all-time points, PTX concentrations determined in the glioma section followed the order: Pep-NP-PTX > NP-PTX > Taxol^®^ (Fig. 6A). 0.5, 1 and 4 h after administration, PTX level in the glioma section of Pep-NP-PTX group were 1.98, 1.91 and 1.53-fold over that of NP-PTX group, and 3.22, 2.58 and 1.32-fold over that of Taxol^®^ group, respectively, suggesting that more PTX accumulated in the glioma section. These results were in agreement with our previous observations that the highest amount of coumarin-6 was found in the glioma section of intracranial glioma bearing mice when loaded in Pep-NP^17^. The concentrations of PTX in the plasma had no significant difference between the Taxol^®^, NP-PTX and Pep-NP-PTX at any time points post-injection (Fig. 6B).

### *In vivo* anti-glioma efficacy

**Figure 7:**
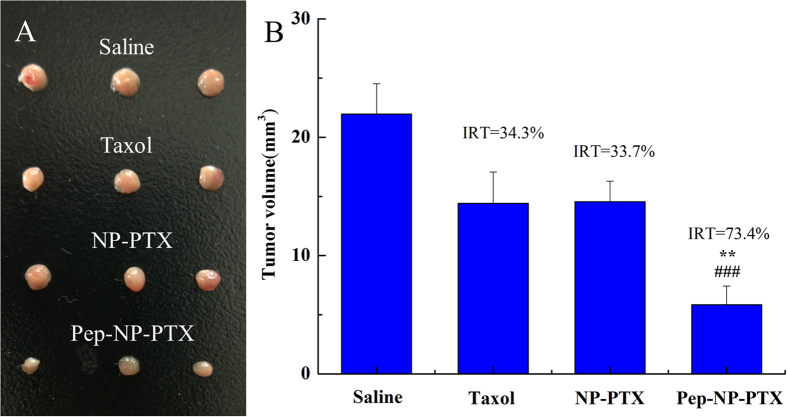
Photograph of glioma from each treatment group excised on day 14 post-implantation (**A**); the glioma volume of each treatment group at the time of sacrifice. IRT: inhibition ratio of tumor (%) (**B**). **p < 0.01 significantly higher than that of Taxol^®^, ###p < 0.001 significantly higher than that of NP-PTX.

The glioma-bearing mice were intravenously administered with Taxol^®^, NP-PTX, Pep-NP-PTX (PTX dose of 10 mg/kg) every 2 days for four consecutive injections with glioma collected until the fourteenth day. As shown in Fig. 7A, the glioma sizes received the PTX formulations were all notably smaller than that of the saline group. Based on tumor volume, the inhibition ratio of tumor of Pep-NP-PTX, NP-PTX and Taxol^®^ were calculated to be 73.4%, 33.7% and 34.3%, respectively (Fig. 7B).

**Figure 8:**
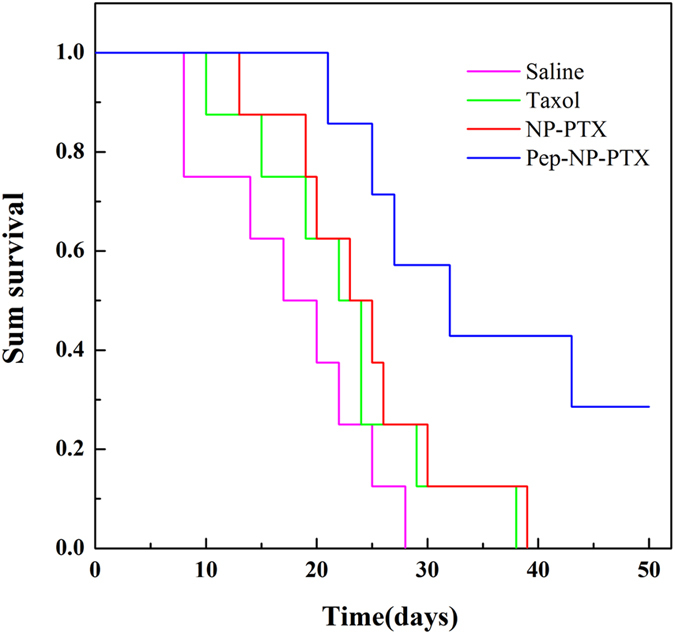
Kaplan-Meier survival curves of C6 glioma-bearing mice treated with different PTX formulations at a dose of 10 mg/kg PTX on day 2, 4, 6 and 8 post implantation.

The anti-glioma efficacy was also evaluated by measuring the survival time of the intracranial glioma-bearing mice treated with the different PTX formulations (Fig. 8 and Table 3). The medium survival time of Pep-NP-PTX group was 32 days, suggesting that Pep-NP-PTX significantly prolonged animal survival time when compared with Saline (17 days), Taxol^®^ (22 days) and NP-PTX (23 days). These results indicated that Pep-1 conjugated PEG-PLGA nanoparticles constructed in this study showed a desirable therapeutic effect, which could offer a potential drug delivery system for glioma treatment.

### *In vivo* safety evaluation

**Figure 9:**
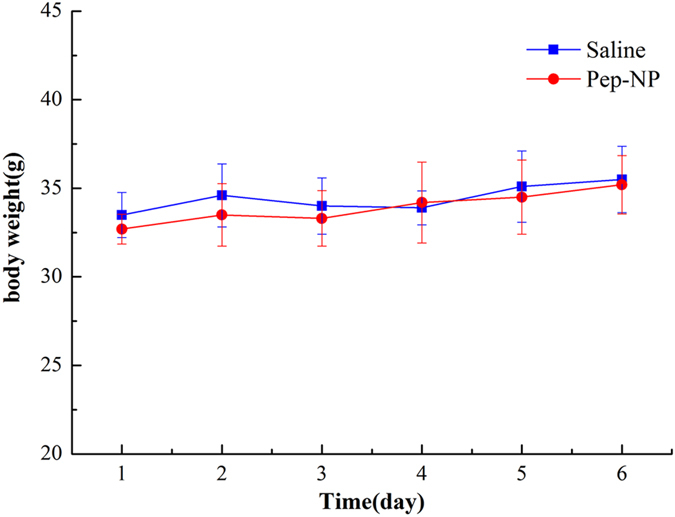
Change in body weight of animals as a function of time in healthy ICR mice. Mice were injected i.v. administration with 100 mg/kg blank Pep-NP or negative control (Saline) for 6 days, one dose per day (n = 5).

**Figure 10:**
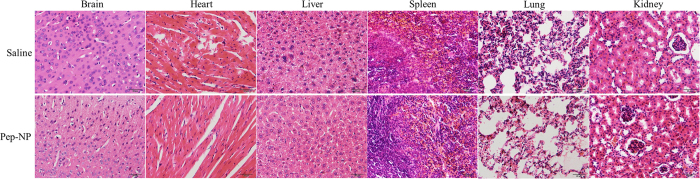
Histochemistry analysis of organs (brain, heart, liver, spleen, lung and kidney) stained with H&E of ICR mice 24 h after i.v. administration of 100 mg/kg blank Pep-NP for 6 days, one dose per day, and saline as control.

The systemic toxicity of blank Pep-NP was evaluated in healthy mice. Compared with the saline group, no deaths and obvious body weight loss were observed in all test groups during the study period (Fig. 9). The tissue sections of heart, liver, spleen, lung, kidney and brain stained with H&E showed no any apparent change in cellular structure and no necrosis, congestion or hydropic degeneration was observed compared with the saline group (Fig. 10). Moreover, there was no significant difference about the serum aspartate transaminase (AST), alanine transaminase (ALT), urea nitrogen (BUN) and creatinine levels between Pep-NP and saline group (Table 4), indicating that no inflammatory reactions occurred in these tissues. Taken together, our results exhibited that intravenous successive administration of 100 mg/kg Pep-NP for 6 days did not cause systemic toxicity.

## Discussion

Treatment of GBM, the most frequent primary central nervous system tumor, is one of the most challenging problems^18^. Surgical resection generally fails to control progression of the glioma, and recurrence is practically inevitable^19^, due to its diffuse invasion of the surrounding normal tissue. In addition, the currently available chemotherapy is less than optimal for glioma treatment, mainly owing to the delivery problems. Most of the therapeutic drugs can’t be delivered to the glioma directly for the existence of BTB. It acts as a physical and biological barrier to protect the glioma from chemotherapeutic agents to ensure an optimal environment for glioma.

In recent years, the emphasis for treatment of glioma has been the application of receptor-mediated transcytosis for the targeting delivery of drug into the glioma. It provides an opportunity for active targeting to overcome the poor permeation of BTB, particularly when the receptor is up-regulated under the diseased condition. Therefore, receptor-mediated transcytosis is also known as the molecular Trojan horse approach. ANG1005^20^, a conjugation complex of PTX with a receptor-targeting peptide (Angiopep-2), exhibited an efficient therapeutic strategy for increasing the potency of PTX for GBM^21^ and breast cancer^22^. Although this approach was originally applied to molecular or macromolecular cargos, it has now been extended to nanoparticulate drug delivery system with the same level of success^23^,^24^,^25^,^26^.

Among these receptors, IL-13Rα2 has attracted increasing interest for its potential role in tumor-specific therapeutics. It has been found to be significantly up-regulated in a number of human tumors, including glioblastomas^27^,^28^ and ovarian carcinomas^29^. IL-13 has the ability to form a second complex with its high-affinity receptor, IL-13Rα2. However, the use of endogenous ligands as a target vector may compete for binding sites due to the very high concentration of endogenous ligands in the circulation. Pep-1, isolated by a cyclic disulphide-constrained heptapeptide phages display library, showed high specificity to IL-13Rα2 surprisingly^30^. In the previous study, we developed a glioma targeting system by courmarin-6-labeled PEG-PLGA copolymer nanoparticles modified with Pep-1^17^. The Pep-NP exhibited enhanced uptake by C6 glioma cells *in vitro* and accumulation in glioma-bearing brain *in vivo* through IL-13Rα2 mediated endocytosis.

In this study, we constructed a targeting nanoparticle drug delivery system by conjugating with Pep-1, which transported PTX for glioma treatment. It is well known that the rapid proliferation of cancer cells leads to a leaky vasculature in the tumor rendering it more permeable, which is better known as the EPR effect^31^. The targeting nanoparticles were prepared by emulsion/evaporation method, and Pep-1 peptide was functionalized to nanoparticle via a maleimide-thiol coupling reaction. Both of the developed NP and Pep-NP were less than 100 nm. These nanoparticles might be suitable for tumor drug delivery as this size range was reported to exhibit a prolonged blood circulation and a relatively low rate of mononuclear phagocyte system (MPS) uptake^32^.

In addition, the resulting NP and Pep-NP showed a similar size distribution and zeta potential, indicating that the nanoparticles might have similar systemic pharmacokinetic behavior (Fig. 6B).

*In vitro* PTX release study showed similar biphases release pattern of the nanoparticles formulations. In the first 6 h, a burst release was obtained. Ten hours later, a mild, sustained and under controlled release was presented. The initial faster release was believed to be derived from the agents that located at the outer layer of the particles while the later slower one from that incorporated into the nanoparticle core and released in a prolonged way along with the erosion or degradation of the matrix^33^,^34^. No significant differences were detected between NP-PTX and Pep-NP-PTX in both pH 7.4 and pH 5.0, suggested that the modification of Pep-1 did not change the release pattern of the nanoparticles.

From *in vitro* cell cytotoxicity study, blank NP and Pep-NP did not show obvious cytotoxicity at all concentrations points, whereas the cytotoxicity of Cremophor EL was higher than that of nanoparticles at concentrations ranged from 10 μg/mL to 1000 μg/mL. This showed that PEG-PLGA block copolymer nanoparticles were one of the better drug delivery carrier with good biocompatibility.

All the PTX formulations blocked the C6 proliferation in a concentration-dependent manner (Fig. 4B). *In vitro* cell uptake study results showed that the concentration of PTX in Pep-NP group was much higher than Taxol^®^ group (p < 0.001) and NP group (p < 0.001) at each concentration point (Fig. 3). The elevated association of Pep-NP also resulted in stronger anti-proliferative following encapsulation of PTX in C6 cells. Hence, the IC_50_ of Pep-NP-PTX was 2.39 times lower than that of NP-PTX, which we believed was contributed by the enhanced cellular association of the nanoparticles following the modification with Pep-1. Although the IC_50_ of Taxol^®^ was lower than Pep-NP-PTX, it may be due to the combined effects of PTX and Cremophor EL. Therefore, we believed that the enhanced cytotoxicity of Pep-NP-PTX mainly attributed to the enhanced cellular uptake facilitated by Pep-1 and IL-13Rα2 interaction.

Based on the biodistribution results in previous study^17^, *in vivo* NIR imaging investigation was performed to further evaluate the glioma targeting efficacy of Pep-NP. In fact, during the glioma occurrence and development, the blood-brain barrier endothelial cells may be affected and blood-brain barrier tight junction’s ultrastructure can be destroyed to some extent. The higher the level of brain glioma, the greater degree of damage of blood-brain barrier tight junction integrity occured^35^,^36^,^37^. In our study, the intracranial tumor xenograft model was established by inoculation of C6 14 days, which reached the most advanced stage glioma. Therefore, blood-brain barrier is not the main obstacle for Pep-NP-PTX penetration into glioma in our used model. As shown in Fig. 5C, NP exhibited a modest tumor accumulation after 24 h due to the EPR effect. But Pep-NP group exhibited much stronger fluorescence intensity in the glioma site at all-time points when compared with that of unmodified NP. Although *ex vivo* liver and spleen exhibited intensive fluorescence, *ex vivo* brain imaging in Pep-NP group showed stronger fluorescence signal in glioma site and was in good consistent with the *in vivo* imaging result. The results indicated that the modification of Pep-1 enhanced nanoparticle accumulation in the glioma section through IL-13Rα2 mediated endocytosis than unmodified NP which depended on limited EPR effects to get into tumor site.

Biodistribution study in glioma-bearing mice showed that PTX concentration detected in the glioma section at all-time points followed the order: Pep-NP-PTX > NP-PTX > Taxol^®^ (Fig. 6A). Due to the favorable EPR effects, NP-PTX showed higher drug accumulation in the glioma site compared with Taxol^®^. Pep-NP-PTX, relied on Pep-1 which has high affinity with IL-13Rα2 overexpressed on the surface of C6 glioma cells, achieved the highest PTX accumulation in the glioma site. This result illustrated that Pep-NP-PTX exhibited a desirable glioma biodistribution profile with significantly increased PTX delivery *in vivo* glioma region through IL-13Rα2 mediated endocytosis.

For evaluating the anti-tumor efficacy *in vivo*, the glioma-bearing mice were treated with saline, Taxol^®^, NP-PTX and Pep-NP-PTX every two days for four injections at PTX dose 10 mg/kg. The tumor size of the PTX-treated groups was all notably smaller than that of the saline group. In addition, Pep-NP-PTX showed the strongest ability to inhibit tumor growth (IRT = 73.4%), in comparison with Taxol^®^ (p < 0.01) and NP-PTX (p < 0.001). Moreover, an obvious prolonged survival was achieved in those glioma-bearing mice treated with Pep-NP-PTX, the medium survival time 32 days, significantly higher than NP (p < 0.05), Taxol^®^ (p < 0.05) and saline (p < 0.05). No significant difference was found between the NP with Taxol^®^ and saline groups. These results offered the robust evidence for Pep-1 modified nanoparticles mediated targeted therapeutic benefits of glioma.

It is well known that most of the intravenously injected nanoparticles are taken up and eliminated by mononuclear phagocyte system (MPS), including liver and kidney tissue^38^. Thus, an increase in biochemical parameters including AST, ALT, blood urea nitrogen and creatinine could reflect acute inflammation in liver and kidney. In our study, there were no such inflammatory reactions caused by Pep-NP, indicating that intravenous successive administration of Pep-NP did not cause acute toxicity. Further studies will be performed to illuminate the long-term toxic effects of the Pep-NP.

## Conclusions

In this study, we proposed PEG-PLGA nanoparticles modified with Pep-1 and loaded with PTX as an effective drug delivery system through IL-13Rα2 mediated endocytosis in mediating PTX transport for the treatment of GBM. Compared with NP-PTX, Pep-NP-PTX (p < 0.001) exhibited an enhanced uptake in C6 cells, thus inducing a strengthened anti-proliferation effect with an IC_50_ of 146 ng/ml. It also exhibited a favorable brain biodistribution with increased PTX delivery in the glioma site. More importantly, compared with that of Taxol^®^ and NP-PTX, Pep-NP-PTX showed desirable anti-glioma efficacy with significantly enhanced the survival of glioma-bearing mice. Systemic safety tests showed no acute toxicity to hematological system, heart, liver, spleen, lung, kidney and brain in mice after intravenous administration of blank Pep-NP per day for six days. Taken together, these results indicated that IL-13Rα2 could be exploited as a prospective receptor and PEG-PLGA nanoparticle modified with the high affinity and specificity ligand Pep-1 was a potential targeting drug delivery system for glioma treatment *via* improving the penetration across BTB.

## Materials and methods

### Materials

Methoxyl poly(ethylene glycol)-co-poly(D,L-lactic-co-glycolic acid) copolymer (MePEG-PLGA, 40 KDa) and Maleimidyl-poly(ethylene glycol)-co-poly(D,L-lactic- co-glycolic acid) copolymer (Male-PEG-PLGA, 41.5 KDa) were synthesized by the ring opening polymerization as described before^39^. PTX was purchased from Zelang Medical Technology Co., Ltd. (Nanjing, China). 3-(4, 5-dimethylthiazol-2-yl)-2, 5-diphenyltetrazolium bromide (MTT) was purchased from Beyotime Biotechnology Co., Ltd. (Nantong, China). Dir was obtained from Biotium (Invitrogen, USA). BCA kit and TritonX-100 were purchased from Beyotime Biotechnology Co., Ltd. (Nantong, China). Penicillin-streptomycin, RPMI 1640 medium, fetal bovine serum (FBS) and 0.25% (w/v) trypsin solution were purchased from Gibco BRL (Gaithersberg, MD, USA). All the other solvents were analytical grade.

### Cell line

The C6 cell line was obtained from Institute of Biochemistry and Cell Biology, Shanghai Institutes for Biological Sciences, Chinese Academy of Sciences (Shanghai, China). Cell line was cultured in RPMI 1640 medium, supplemented with 10% FBS, 1% penicillin and 100 mg/mL streptomycin sulfate. Cells were cultured in incubators maintained at 37 °C with 5% CO_2_. All experiments were performed in the logarithmic phase of cell growth.

### Animals

Male ICR mice (male, 4–5 weeks, 18 ± 2 g) and male Balb/c mice (male, 4–5 weeks, 18 ± 2 g) were supplied by Department of Experimental Animals, Nanjing Medical University (Nanjing, China) and maintained under standard housing conditions. All animal experiments were performed in accordance with protocols evaluated and approved by the ethics committee of Nanjing Medical University.

### Preparation of Pep-NP-PTX

Pep-1 conjugated PEG-PLGA (Pep-PEG-PLGA) was synthetized by our previously-described method^17^. Pep-1 conjugated nanoparticle was prepared through emulsion/solvent evaporation method. Briefly, 19 mg MePEG-PLGA copolymer, 2 mg Pep-PEG-PLGA and 1 mg PTX were dissolved in 1 mL ethyl acetate, which was then added into 2 mL 1% (w/v) poloxamer 188 aqueous solution. The mixture was sonicated using a probe sonicator (Xin Zhi Biotechnology Co., Ltd., China) for 5 min at 190 W output. The primary emulsion formed was added drop-wisely into 10 mL 0.5% (w/v) poloxamer 188 aqueous solution under moderate stirring. Ethyl acetate was evaporated at 40 °C using rotary evaporator. The resultant bluish solution was filtrated through 0.45 μm and 0.22 μm cellulose acetate filter membrane to remove the aggregates. The nanoparticles were concentrated by ultrafiltration (10 kd MWCO Millipore, USA) and washed twice to remove excessive emulsifier. Finally, the Pep-NP-PTX suspension was kept at 4 °C for further use.

The preparation of DiR-labeled nanoparticles was prepared with the same procedure as NP except 0.2 mg DiR were dissolved in the ethyl acetate solution.

### Particle size and zeta potential of Pep-NP-PTX

The morphology of Pep-NP-PTX was investigated using transmission electron microscope (TEM). The particle size and zeta potential of the nanoparticles were determined by dynamic light scattering (DLS) (Zs90, Malvern, U.K.).

### Encapsulation efficiency and loading capacity of Pep-NP-PTX

The amount of PTX encapsulated in the nanoparticles was measured by HPLC method (LC-10AT, SHIMADZU, Japan). A reverse phase C-18 Ultrasphere ODS column (150×4.6 mm, 5 mm, Thermo Fisher, USA) was used. The mobile phase consisted of acetonitrile and water (47:53, v/v). The wavelength of detection was 227 nm. The flow rate was set at 1.0 mL/min, and the column was maintained at 30 °C. The retention time of PTX was about 7.9 min. The calibration curve was linear in the range of 0.1–100 μg/mL with a correlation coefficient of R^2^ = 0.9998.

To determine the EE and LC of Pep-NP-PTX, the concentration of PTX in samples was dissolved in acetonitrile and analyzed by HPLC as described. The EE% and LC% were calculated as indicated below (n = 3).


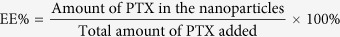



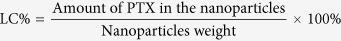


### *In vitro* release

The *in vitro* release behavior of PTX from nanoparticles was monitored in PBS (0.04 M, pH 7.4) or HAc-NaAc buffer (0.04 M, pH 5.0) containing 0.1% (w/v) Tween-80 at 37 °C by dialysis method. Briefly, PTX-loaded NP and Pep-NP were dispersed in 1 mL of mediator solution, and then sealed in a dialysis bag (MWCO 7000). The bag was immersed in 40 mL of the same mediator solution. A portion of 0.4 mL dialysate was taken out at various time and replaced by 0.4 mL fresh buffer solution. The concentration of PTX in samples was determined by HPLC as described above with correction for the volume replacement.

### Cellular uptake of PTX-loaded Pep-NP

For quantitative experiment, the cells were seeded into a 24-well plate at the density of 1×10^5^ cells per well. After 24 h incubation, the cells were incubated with nanoparticles at different concentrations at 37 °C, respectively. 2 h later, Taxol^®^, NP-PTX and Pep-NP-PTX were added into wells at the PTX concentration ranged from 10 to 60 μg/ml, respectively. At the end of the incubation period, the samples were removed from the wells and the cell monolayers were washed with cold PBS. The cells were lysed by 400 μL of 1% TritonX-100 per well for 10 min. An aliquot of the cell lysate from each well was used to determine the total cell protein content using the BCA protein assay. The concentration of PTX in samples was determined by HPLC as described above.

### Cell cytotoxicity

The cell cytotoxicity of Pep-NP-PTX on C6 cells was evaluated by using the MTT assay. C6 cells were seeded into 96-well plates at the density of 5000 cells/well and incubated at 37 °C in a 5% CO_2_ atmosphere. 24 h later, Taxol^®^, NP-PTX and Pep-NP-PTX were added into wells at the PTX concentration ranged from 0.01 to 20 μg/ml, respectively. After 72 h incubation, 20 μL MTT was added into each well and incubated for 4 h. Then the unreacted dye was removed and 200 μl of DMSO was added to each well to dissolve the dark blue crystal. Finally, the optical density was measured by microplate reader at wavelength of 490 nm. Cells without exposure to the PTX formulations were used as control.

The cell cytotoxicity of blank nanoparticles was evaluated also by the same me-thod with the concentration ranged from 0.1 to 1000 μg/ml.

### *In vivo* imaging

The intracranial tumor xenograft model was established by inoculation of C6 cells (5.0 × 10^5^ cells suspended in 5 μL PBS) into the the right striatum (1.8 mm lateral to the bregma and 3 mm of depth) of male Balb/c mice. The release of Dir from NPs is very low, indicating that Dir could be accurate fluorescence probes for NP behavior *in vivo*, and the fluorescence signals detected in organs well represented the distribution of the NPs^40^. After cultured for 14 days, the glioma-bearing mice were injected with Dir-loaded NP and Pep-NP at the dose of 0.8 mg/kg Dir, the concentration of Dir was measured by fluorescent spectrophotometer.

The fluorescent images were acquired at 3 and 24 h after injection using an *in vivo* imaging system (Caliper, USA). After 24 h post-injection, the mice were sacrificed and the fluorescence of tissues (including heart, liver, spleen, lung, kidney and brain) were visualized under the *in vitro* imaging system.

### Biodistribution of Pep-NP-PTX in intracranial glioma-bearing mice

Forty five glioma-bearing ICR mice were randomly divided into three groups and intravenously injected with Taxol^®^, NP-PTX and Pep-NP-PTX at the dose of 8 mg/kg PTX, respectively. At different time points (0.5, 1 and 4 h, n = 5 at each time point) after injection, brain and glioma of the mice were collected. The tissues were homogenized in 0.9% sodium chloride solution with 1% TritonX-100 after the weight measurement. The supernatant was obtained after centrifugation.

For the determination of PTX, 100 μL supernatant of tissue homogenates (or 100 μL plasma) were mixed with 100 μL methanol containing 100 ng/mL docetaxel (internal standard). Then 1 mL ether was added to extract the PTX and docetaxel. After dried the organic phase under N_2_, the residue was dissolved with 200 μL 80% methanol solution and analyzed by HPLC-mass spectrometry (Agilent 1200 LC-MS, USA).

### *In vivo* anti-glioma efficacy

*In vivo* tumor growth inhibition was performed to evaluate the anti-glioma efficacy of the constructed formulations. Glioma-bearing ICR mice model was established as described above. Two days after the implantation, the mice were randomly divided into four groups (3 mice per group) and intravenously administered with saline, Taxol^®^, NP-PTX and Pep-NP-PTX at the dose of 10 mg/kg PTX, respectively. The treatment was repeated every other day for four injections. Fourteen days later, the mice were sacrificed with tumor collected and tumor volume was calculated with the formula: π/6 × larger diameter × (smaller diameter)^2^,^41^.

The anti-tumor efficacy of the formulations was also evaluated on intracranial glioma-bearing mice by measuring their survival time. Two days after C6 cells injection, the ICR mice were randomly divided into 4 groups (8 mice per group): saline group, Taxol^®^ group, NP-PTX group and Pep-NP-PTX group. Each group was intravenously administered at the dose of 10 mg/kg PTX. The treatment was repeated every other day for four injections. The survival data were presented as Kaplan-Meier plots and analyzed with a log-rank test.

### *In vivo* safety evaluation

Ten male ICR mice were randomly divided into two groups (n = 5). Each group received an intravenous injection of blank Pep-NP (100 mg/kg) or saline per day for six injections. The body weight was monitored each day. Blood sample and tissues (heart, liver, spleen, lung, kidney and brain) were collected at 24 h after the last administration for hematologic and histochemistry analysis. The AST, ALT, BUN and creatinine levels were assayed using Hitachi 7080 Chemistry Analyzer (Hitachi Ltd., Japan). The tissues were fixed with paraformaldehyde for 24 h and embedded in paraffin. Each brain was cut into 5 μm by parafin section, processed for routine hematoxylin and eosin (H&E) staining, and then visualized under fluorescent microscope (Imager A1, Zeiss, Germany).

### Statistical analysis

All the results were expressed as mean ± standard deviation (SD). Statistical analysis was performed with SPSS 20.0 software. Statistical analysis was used one-way ANOVA test. Differences were considered significant when *p < 0.05, **p < 0.01, ***p < 0.001, respectively.

## Additional Information

**How to cite this article**: Wang, B. *et al.* Improved anti-glioblastoma efficacy by IL-13Rα2 mediated copolymer nanoparticles loaded with paclitaxel. *Sci. Rep.* **5**, 16589; doi: 10.1038/srep16589 (2015).

## Figures and Tables

**Table 1:** Characterization of PTX-loaded NP and Pep-NP.

Formulation	Particles size (nm)	Polydispersity index (PDI)	Zeta Potential (mV)	LC(%)	EE(%)
NP-PTX	91.23 ± 1.56	0.122 ± 0.033	−35.2 ± 2.15	3.93 ± 0.096	79.83 ± 1.57
Pep-NP-PTX	95.78 ± 2.37	0.137 ± 0.047	−34.5 ± 1.74	3.77 ± 0.104	77.27 ± 0.95

**Table 2:** IC_50_ of PTX formulations against C6 cell lines (n = 5).

Formulation	Taxol^®^	NP	Pep-NP
IC_50_(μg/mL)	0.070 ± 0.002	0.349 ± 0.012	0.146 ± 0.003

**Table 3:** *In vivo* effects of PTX formulations on intracranial C6 glioma mice model (n = 7 or 8).

Groups	Dose (mg/kg)	MST (days)	Median (days)	Compare with Saline	Compare with Taxol^®^	Compare with NP
Saline	—	17.7 ± 2.6	17	—	—	—
Taxol^®^	10	22.6 ± 3.0	22	p > 0.05	—	—
NP-PTX	10	24.4 ± 2.7	23	—	p > 0.05	—
Pep-NP-PTX	10	35.4 ± 4.2	32	**	*	*

MST: mean survival time

**p < 0.01, *p < 0.05 of log-rank analysis

**Table 4:** Mice serum level of biochemical variables after intravenous treatment with Pep-NP at concentration of 100 mg/kg for 6 days (n = 4).

Groups	Creatinine (μmol/L)	BUN (mmol/L)	ALT (U/L)	AST (U/L)
Pep-NP	5.44 ± 0.28	5.32 ± 0.77	8.55 ± 2.20	10.12 ± 2.07
Saline	5.82 ± 0.37	5.86 ± 0.81	10.71 ± 2.22	12.06 ± 1.28

Values are the means ± SD, there was no significant difference of the above parameters between blank Pep-NP group and saline control (p > 0.05). BUN: blood urea nitrogen; ALT, serum alanine aminotransferase; AST: aspartate aminotransferase.
